# Equalizing the In-Ear Acoustic Response of Piezoelectric MEMS Loudspeakers Through Inverse Transducer Modeling

**DOI:** 10.3390/mi16060655

**Published:** 2025-05-29

**Authors:** Oliviero Massi, Riccardo Giampiccolo, Alberto Bernardini

**Affiliations:** Dipartimento di Elettronica, Informazione e Bioingegneria (DEIB), Politecnico di Milano, Piazza L. Da Vinci 32, 20133 Milano, Italy; oliviero.massi@polimi.it (O.M.); alberto.bernardini@polimi.it (A.B.)

**Keywords:** MEMS loudspeakers, piezoelectric transducers, equalization, inverse systems

## Abstract

Micro-Electro-Mechanical Systems (MEMS) loudspeakers are attracting growing interest as alternatives to conventional miniature transducers for in-ear audio applications. However, their practical deployment is often hindered by pronounced resonances in their frequency response, caused by the mechanical and acoustic characteristics of the device structure. To mitigate these limitations, we present a model-based digital signal equalization approach that leverages a circuit equivalent model of the considered MEMS loudspeaker. The method relies on constructing an inverse circuital model based on the nullor, which is implemented in the discrete-time domain using Wave Digital Filters (WDFs). This inverse system is employed to pre-process the input voltage signal, effectively compensating for the transducer frequency response. The experimental results demonstrate that the proposed method significantly flattens the Sound Pressure Level (SPL) over the 100 Hz-10 kHz frequency range, with a maximum deviation from the target flat frequency response of below 5 dB.

## 1. Introduction

Micro-Electro-Mechanical Systems (MEMS) loudspeakers represent a rapidly advancing class of miniature transducers designed for audio reproduction in compact consumer electronics [1]. Unlike conventional electrodynamic and balanced armature micro-speakers, which offer only limited room for improvement in the light of increasing demands for wireless connectivity, device miniaturization, and sustainability, MEMS technologies offer a fundamentally different paradigm. Leveraging established semiconductor manufacturing processes, MEMS loudspeakers enable extreme miniaturization, high on-chip integration density, low power consumption, and scalable, cost-effective production [2,3], positioning them as a promising solution for next-generation audio systems. In recent years, numerous research efforts have been focused on the development of novel designs and structures for MEMS loudspeakers [4,5,6,7], aimed at improving their acoustic performance especially in terms of the Sound Pressure Level (SPL). Piezoelectric actuation has emerged as the dominant technology in this context owing to its ability to produce strong mechanical forces at relatively low actuation voltages [5].

Despite these advancements, the MEMS loudspeakers used for in-ear applications often exhibit highly irregular frequency responses [6,7], typically characterized by sharp resonant peaks. These artifacts stem from the mechanical resonances in the diaphragm and its supporting structures, as well as from the acoustic resonances introduced by the surrounding cavities, either within the loudspeaker package or in the ear canal itself [8]. This behavior is highly undesirable, as it introduces spectral coloration, compromises audio fidelity, and can contribute to listener fatigue [9]. To ensure the practical usability of these MEMS loudspeakers in real-world applications, the use of appropriate signal pre-processing strategies should be taken into account. Among these, equalization techniques that effectively compensate for the transducer’s resonant behavior are essential for achieving a flat frequency response with minimal perceived spectral coloration.

Loudspeaker equalization refers to the process of shaping the input signal to counteract the non-ideal frequency response of a loudspeaker, with the goal of achieving a desired frequency response in the audio bandwidth [10]. In the context of macroscale electrodynamic loudspeakers, a wide range of Digital Signal Processing (DSP) equalization techniques have been proposed and investigated, ranging from simple graphic and parametric equalizers to more advanced techniques based on digital filter design, which include Finite Impulse Response (FIR) filter design methods as well as Infinite Impulse Response (IIR) filter techniques [10,11,12]. In the latter category of methods, model-based equalization methods have gained significant attention due to their ability to provide accurate and physically interpretable correction filters [13,14,15]. These approaches generally rely on a description of the loudspeaker’s behavior using Lumped-Element Models (LEMs) [15], which provide a lightweight yet physically meaningful characterization of system dynamics. Once such a model is identified, either analytically or from experimental data, we define an inverse system in the digital domain to effectively correct the non-ideal loudspeaker frequency response in a pre-processing stage. Notably, loudspeaker equalization can be readily interpreted as a specific instance of *loudspeaker virtualization* algorithms, wherein the goal is to make one transducer emulate the acoustic behavior of another reference device [16]. Within this framework, the inverse system serves to cancel out the inherent response of the physical loudspeaker. To achieve the desired target behavior, an additional direct model may be inserted upstream in the signal chain, preceding the inverse system. This configuration, known as the *Direct–Inverse–Direct Chain* (DIDC) processing structure, consists of two digital processing stages: the target direct system that defines the desired response and the inverse system. These are followed by the physical transducer itself, forming the final stage that reproduces the desired audio output [15,16].

In recent years, several works have extended lumped-element modeling techniques to the domain of MEMS loudspeakers, proposing equivalent circuit model representations tailored to the actuation, structural, and acoustic properties of such devices [8,17,18,19]. These models have proven valuable for understanding the electromechanical behavior as well as guiding the design and optimization of MEMS transducers. However, despite their increasing accuracy and availability, such models have not yet been systematically utilized for the development of DSP techniques aimed at correcting loudspeakers’ acoustic response. To date, the MEMS loudspeaker equalization task has been primarily limited to the direct design of FIR filters based on the inverse transfer function of the loudspeaker superimposed with a possible target frequency response [4,7].

In this manuscript, we address the problem of in-ear acoustic response equalization for piezoelectric MEMS loudspeakers by explicitly incorporating the discrete-time simulation of LEMs into the signal processing chain. With the purpose of achieving a flat frequency response, we devise a virtualization chain based on the inverse model of a MEMS loudspeaker’s linear equivalent circuit model. The design of the inverse system relies on Leuciuc’s theorem [20], reworded in [16,21], and it is based on the addition of a theoretical two-port element to the direct system, known as a *nullor*. The resulting inverse system is implemented and efficiently simulated in the discrete-time domain using Wave Digital Filter (WDF) principles [22,23,24,25]. The proposed signal processing chain is validated through experimental SPL measurements, which demonstrate its ability to compensate for the strong resonant behavior observed for the considered MEMS loudspeaker. The results highlight a substantial improvement in the frequency response flatness and demonstrate the potential of the approach for enhancing the audio fidelity in in-ear applications.

The remainder of this manuscript is organized as follows: Section 2 introduces the equivalent circuit model of the MEMS loudspeaker under study. Section 3 details the design of the equivalent circuit model of the inverse MEMS loudspeaker for loudspeaker equalization, along with its discrete-time implementation. The experimental results are presented and discussed in Section 4. Finally, Section 5 concludes this manuscript.

## 2. MEMS Loudspeaker Equivalent Circuit Model

**Figure 1:**
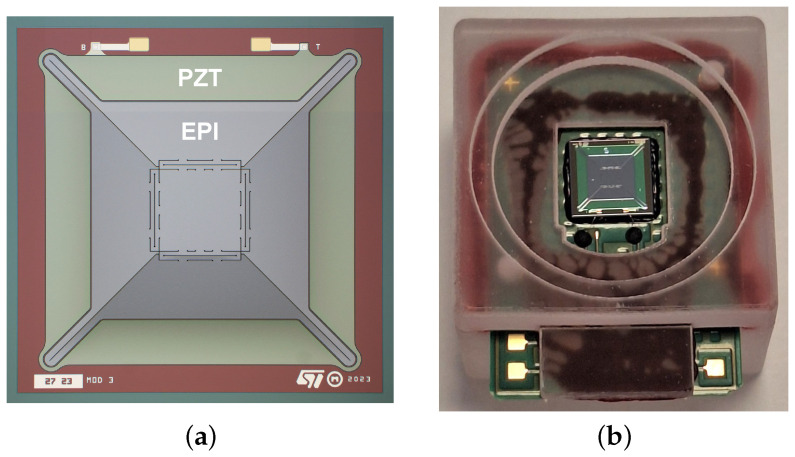
(**a**) Optical microscope image of the fabricated MEMS loudspeaker showing the PZT stack distribution. The MEMS device has a total footprint of $4.5\times 4.5\;{\mathrm{mm}}^{2}$. (**b**) The considered MEMS loudspeaker mounted into the thermoplastic packaging, with a 1 cm^3^ back chamber.

The MEMS loudspeaker considered in this work is fabricated by STMicroelectronics and represents an enhanced version of the design originally introduced in [5] for in-ear audio applications. Maintaining the same total footprint of $4.5\times 4.5\;{\mathrm{mm}}^{2}$, the loudspeaker’s mechanical structure consists of four trapezoidal actuators symmetrically linked to a central squared piston via newly designed suspension springs, as illustrated in Figure 1a. The device is fabricated with a 13µm-thick epitaxial (EPI) silicon layer and a 2µm-thick Lead Zirconate Titanate (PZT) layer. Compared to the previous generation of loudspeakers [5], the amount of deposited piezoelectric material is reduced, halving the static capacitance of the transducer. The internal spacing between the mechanical components is defined by 5µm air gaps. For characterization and testing, the MEMS loudspeaker is assembled on a custom Printable Circuit Board (PCB) and enclosed within a thermoplastic package, as shown in Figure 1b. This package includes a $1\;{\mathrm{cm}}^{3}$ back chamber and a 1mm front adapter, enabling direct coupling to the IEC 60318-4 ear simulator [26], which emulates the in-ear acoustic conditions.

**Figure 2:**
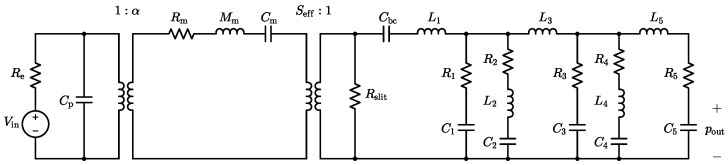
Linear equivalent circuit model of the target piezo-actuated MEMS loudspeaker for in-ear applications.

The considered MEMS loudspeaker, when coupled to the ear simulator, is described by the linear equivalent circuit model depicted in Figure 2. In the electrical domain, the circuit features a voltage generator $V_{\mathrm{in}}$ and a series resistance $R_{e}$, modeling the loudspeaker input driving voltage and the wiring resistance, respectively. The static capacitance of the piezoelectric layer is represented by the capacitor $C_{p}$, with dielectric and leakage losses considered negligible in this analysis. The linear piezoelectric transduction process is modeled with an ideal transformer with turn ratio 1:α, where α defines the electro-mechanical transduction coefficient.

In the mechanical domain, the vibration of the diaphragm is modeled as a single-degree-of-freedom oscillator. In this representation, $R_{m}$ accounts for the damping due to viscous losses, $M_{m}$ reflects the participating mass of the moving components, and $C_{m}$ characterizes their mechanical compliance. The coupling between the mechanical domain and the acoustic domain is described by a second ideal transformer, with its turn ratio set by the effective radiating area of the moving diaphragm $S_{\mathrm{eff}}$.

In the acoustic domain, the acoustic compliance of the $1\;{\mathrm{cm}}^{3}$ back chamber is represented by the capacitor $C_{\mathrm{bc}}$. The resistor $R_{\mathrm{slit}}$ models the acoustic viscous losses introduced by the 5µm air gaps located between the actuating mechanical components, which inhibit the complete acoustic decoupling of the front volume from the back chamber. The parameter values used in the equivalent circuit model are summarized in Table 1. Finally, the model also integrates an equivalent circuit representation of the IEC 60318-4 ear simulator, with the corresponding parameter values listed in Table 2. Three additional damping terms, $R_{1}$, $R_{3}$, and $R_{5}$, are included to improve the agreement between the simulated and measured responses of the loudspeaker system. The acoustic output pressure $p_{\mathrm{out}}$, measured at the ear simulator microphone, corresponds in the electrical analogy to the voltage across capacitor $C_{5}$.

Most of the parameter values listed in Table 1 and Table 2 are directly extrapolated from previous works [8,19], as they remain unaffected by the recent design changes. However, to account for the changes introduced by the updated piezoelectric and mechanical structure, we fine-tune a subset of model parameter values using the optimization framework described in [27]. Through this method, we frame the LEM parameter estimation as a model optimization problem in a supervised learning paradigm, which involves the gradient-based minimization of the discrepancy between the simulated and measured SPL responses. This optimization is selectively applied to the parameters likely impacted by the design evolution, specifically the electro-mechanical transduction coefficient α; the mechanical elements $R_{m}$, $M_{m}$, and $C_{m}$; along with the acoustic damping terms $R_{\mathrm{slit}}$, $R_{1}$, $R_{3}$, and $R_{5}$, while all remaining parameters preserve their original values.

In the next section, the developed linear equivalent circuit model of the MEMS loudspeaker is used as the basis for constructing an inverse system designed to equalize and flatten the device’s frequency response.

## 3. Direct–Inverse–Direct Chain MEMS Loudspeaker Equalization

**Figure 3:**
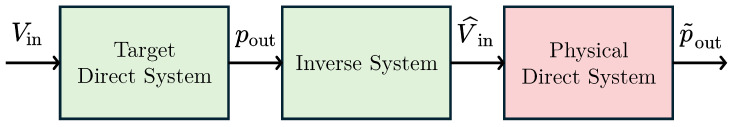
Target-Inverse-Physical Chain processing algorithm.

As discussed in Section 1, loudspeaker equalization can be interpreted as a specific instance of a DIDC-based virtualization algorithm. In the case of actuators, this framework is implemented as a Target-Inverse-Physical Chain (TIPC) [16], illustrated with a block diagram in Figure 3. The green blocks represent the processing elements implemented in the digital domain, while the red block corresponds to the actual physical transducer. In this setup, the signal to be pre-processed is the input voltage ${\hat{V}}_{\mathrm{in}}$ that drives the MEMS loudspeaker, while the goal is to control the behavior of the acoustic pressure ${\tilde{p}}_{\mathrm{out}}$.

In the context of MEMS loudspeaker equalization, where the goal is to achieve a flat frequency response in the audio bandwidth, the *Target Direct System* block is defined to have a flat frequency response, with a magnitude determined by the proportional rescaling factor between the input voltage $V_{\mathrm{in}}$ and the desired output pressure $p_{\mathrm{out}}$, expressed in pascals (Pa). Alternatively, the *Target Direct System* can be omitted entirely, and any desired spectral shaping, such as perceptual equalization, can be applied directly to the $p_{\mathrm{out}}$ signal before the *Inverse System* implementation in the processing chain. The *Inverse System* corresponds to the inverse of the equivalent circuit model of the *Physical Direct System*, which is the transducer itself. Given the desired output pressure behavior $p_{\mathrm{out}}$, the *Inverse System* produces the pre-compensated input voltage ${\hat{V}}_{\mathrm{in}}$ that, when applied to the physical transducer, equalizes its behavior to match the target response.

**Figure 4:**
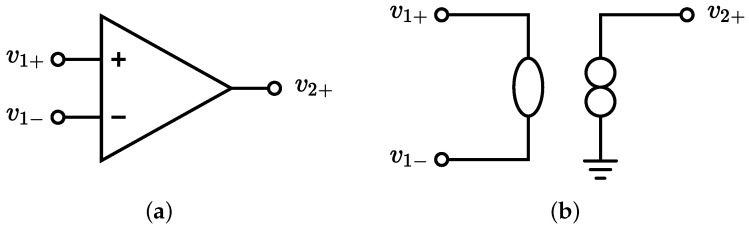
(**a**) Ideal opamp schematic symbol. (**b**) Equivalent nullor-based representation of the same ideal opamp in (**a**).

To implement the *Inverse System* processing block, we begin by constructing the inverse circuital model of the linear equivalent circuit model that characterizes the physical behavior of the MEMS loudspeaker. This inverse model can be directly obtained applying the theorem presented in [20,21] after augmenting the direct system in Figure 2 with a theoretical two-port element called a nullor [28]. A nullor consists of two theoretical one-port elements: a *nullator* (represented as an ellipse), which enforces both zero voltage and zero current at its port, and a *norator* (represented by two circles), which allows arbitrary port variables. Nullors are commonly employed to build ideal macromodels of more complex multiport elements [29]; for example, an ideal operational amplifier (opamp) can be modeled using a nullor, as shown in Figure 4.

**Figure 5:**
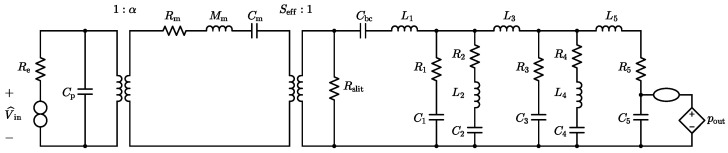
Inverse MEMS loudspeaker equivalent circuit model based on a nullor.

**Figure 6:**
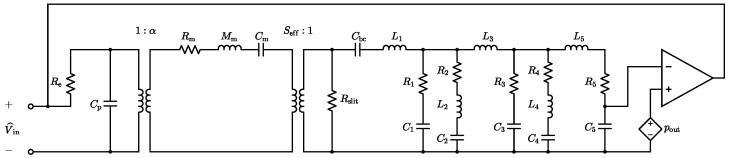
Inverse MEMS loudspeaker equivalent circuit model involving the ideal opamp representation of the nullor.

The nullor-based inverse circuital model is shown in Figure 5. If we consider an ideal operational amplifier, modeled as illustrated in Figure 4, the circuit in Figure 5 can be equivalently redrawn as the configuration in Figure 6. Assuming ideal opamp behavior, the two circuits in Figure 5 and Figure 6 are functionally equivalent. According to the inversion theorem, the voltage ${\hat{V}}_{\mathrm{in}}$ across the norator in the inverse system is equal to the input signal $V_{\mathrm{in}}$ of the direct system, and it is obtained by feeding the inverse system with $p_{\mathrm{out}}$, which is the output of the direct system.

The digital processing chain outlined above implicitly assumes that cascading the discrete-time simulation of the *Inverse System* with the *Physical Direct System* results in an identity operation. In other words, by canceling out the behavior of the physical transducer, the desired output pressure signal $p_{\mathrm{out}}$ can be directly reproduced, meaning that ${\tilde{p}}_{\mathrm{out}}$ coincides with $p_{\mathrm{out}}$. It is important to highlight, however, that the *Inverse System* is derived from a linear model of the MEMS loudspeaker. Consequently, the proposed processing chain is only capable of compensating for the linear dynamics of the device, leaving any nonlinear effects unaddressed.

### 3.1. Inverse System Discrete-Time Simulation

The digital implementation and discrete-time simulation of the *Inverse System*, derived from the MEMS loudspeaker equivalent circuit model through nullor-based inversion, are performed using WDFs due to their advantageous numerical properties, modularity, and computational efficiency [22]. Moreover, they provide an efficient framework for implementing circuits that incorporate nullors [25]. First introduced by Fettweis in the late 1970s [22] to design digital implementations of passive analog circuits, WDFs rely on a port-wise transformation of Kirchhoff variables (voltages and currents) into wave variables (incident and reflected waves). This transformation is commonly defined according to the definition of voltage wave variables

(1) a=v+Zi,b=v−Zi,

where *v* is the port voltage, *i* is the port current, *a* is the incident wave, *b* is the reflected wave, and *Z* is a scalar free-parameter referred to as *port resistance*. In this approach, one-port circuit elements are realized in the Wave Digital (WD) domain as one-port input-output blocks, each defined by a scalar scattering equation as outlined in [24]. In this work, the constitutive equations of linear dynamic elements, such as capacitors and inductors, are discretized using the backward Euler method [24]. All the one-port linear elements are *adapted* by properly setting the corresponding free parameters to remove the delay-free loops [22,24]. The interconnections among the elements are managed by multiport WD junctions, which are characterized by a scattering matrix [23]. As far as the WD implementation of nullors is concerned, they are encompassed into scattering junctions as additional topological constraints, following the methodology discussed in [25]. Being the inverse MEMS loudspeaker equivalent circuit model linear, the resulting WD structure can be solved using traditional implementation techniques [22] in a fully explicit manner, i.e., without the need for iterative solvers.

## 4. Experimental Results

**Figure 7:**
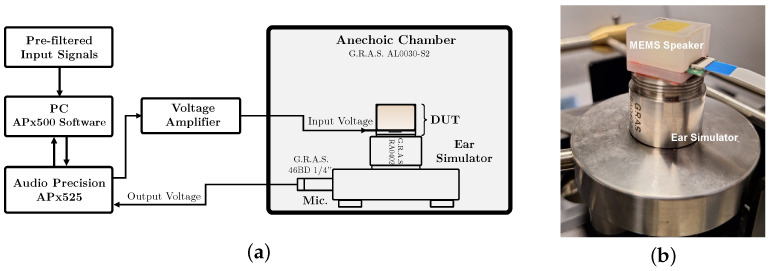
(**a**) Block diagram of the acoustic measurement setup, featuring a G.R.A.S. RA0402 ear simulator and a G.R.A.S. 46BD 1/4” microphone. An Audio Precision APx525 audio analyzer is used to generate both DC and AC signals to drive the MEMS loudspeaker and to record the microphone signal. The analog signals generated by APx525 are amplified by 10× to reach the desired driving level. (**b**) Picture of the MEMS loudspeaker connected to the G.R.A.S. RA0402 ear simulator.

In this section, we validate the proposed equalization processing chain using experimental acoustic measurements of the MEMS loudspeaker under in-ear conditions. The measurement setup is depicted in Figure 7a, and it comprises a G.R.A.S. AL0030-S2 anechoic chamber, a G.R.A.S. RA0402 ear simulator (without the ear canal extension), and a G.R.A.S. 46BD 1/4” microphone. The MEMS loudspeaker, housed in its package, is directly connected to the ear simulator, as shown in Figure 7b. An Audio Precision APx525 audio analyzer is used to generate the analog signals driving the Device Under Test (DUT) and to acquire the microphone signal. The equalized input voltage signals are obtained by implementing the WD structure of the *Inverse System* in MATLAB R2024a. The discrete-time simulation is run at a sampling frequency $f_{s}=96\;\mathrm{kHz}$. The resulting signal, corresponding to ${\hat{V}}_{\mathrm{in}}$, is saved as a .wav file and imported into APx500 v4.4 software via the “Signal Acquisition” measurement feature, enabling the full characterization of the equalized device. During the measurements, all applied voltage signals are summed with a fixed DC bias voltage of 15V to ensure the proper operation of the MEMS loudspeaker.

**Figure 8:**
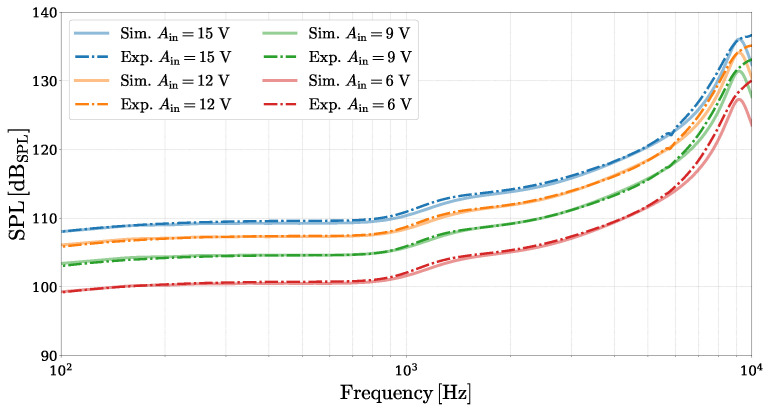
Comparison between SPL curves predicted by the proposed linear equivalent circuit model (solid curves) and the experimental measurements (dash-dotted curves) for different input signal amplitudes.

As a preliminary step, an experimental validation of the MEMS loudspeaker equivalent circuit model proposed in Section 2 is carried out. The model is digitally implemented following WDF principles and simulated in the discrete-time domain, as detailed in Section 3.1. Figure 8 presents a frequency-domain comparison between the SPL obtained from the simulated linear circuit model and the experimental acoustic measurements. The simulated SPL curves are derived by driving the corresponding WD structure with an amplitude-scaled Kronecker delta input signal

(2) $$V_{\mathrm{in}}\left[k\right]=A_{\mathrm{in}}\cdot \delta \left[k\right]\;,$$

where *k* is the discrete-time sample index, and $A_{\mathrm{in}}$ is the input voltage amplitude, directly yielding the system’s pressure impulse response $p_{\mathrm{out}}\left[k\right]$. The frequency-domain SPL is then computed as

(3) $$\mathrm{SPL}\left(\omega_{k}\right)=20\log_{10}\left(\frac{p_{\mathrm{out}}\left(\omega_{k}\right)}{\sqrt{2}p_{\mathrm{ref}}}\right)\;{\mathrm{dB}}_{\mathrm{SPL}}\;,$$

where $p_{\mathrm{out}}\left(\omega_{k}\right)$ is the Discrete Fourier Transform (DFT) of $p_{\mathrm{out}}\left[k\right]$ evaluated at the discrete frequency $\omega_{k}$, and $p_{\mathrm{ref}}=2\times 10^{-5}\;\mathrm{Pa}$ is the reference pressure value. The experimental SPL curves are obtained using an amplitude-scaled logarithmic sine sweep (chirp) signal, where the measured acoustic pressure is deconvolved to retrieve the system’s impulse response [30] and subsequently converted into SPL using (3), as implemented in the APx500 “Continuous Sweep” measurement function. The comparison confirms that the proposed linear model is able to capture the behavior of the MEMS loudspeaker radiating into the ear simulator, with only minor deviations observed near the mechanical resonance peak. This discrepancy is primarily due to the model’s difficulty in precisely matching the experimental resonance frequency, which is close to the upper limit of the excitation sweep and complicates the parameter fine-tuning process.

### 4.1. Equalization Experiments

**Figure 9:**
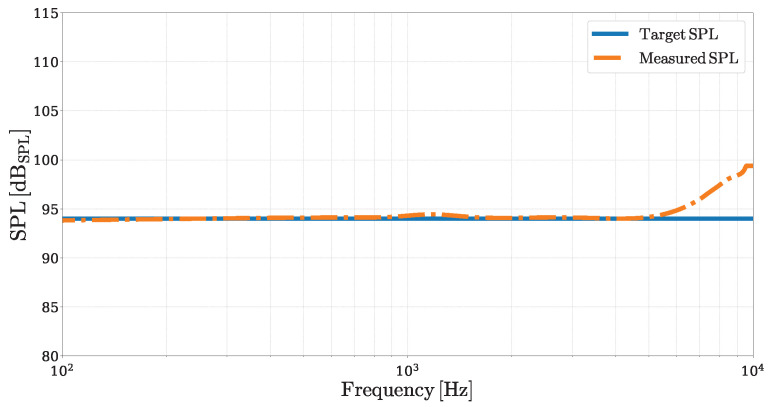
Frequency-domain SPL of the MEMS loudspeaker after $94\;{\mathrm{dB}}_{\mathrm{SPL}}$ flat equalization. The measured SPL (orange dash-dotted curve) is compared to the target SPL (solid blue curve).

As a first equalization experiment, we pre-process the voltage signal ${\hat{V}}_{\mathrm{in}}$ that drives the MEMS loudspeaker, following the scheme illustrated in Figure 3, in order to achieve a flat frequency-domain SPL of $94\;{\mathrm{dB}}_{\mathrm{SPL}}$. A flat SPL of $94\;{\mathrm{dB}}_{\mathrm{SPL}}$ corresponds to a constant pressure amplitude of $\sqrt{2}\;\mathrm{Pa}$ across all considered frequencies $\omega_{k}$. To meet this target, we simulate the *Inverse System* using as input signal $p_{\mathrm{out}}$ a discrete-time logarithmic chirp, defined as in [30], with an amplitude of $\sqrt{2}\;\mathrm{Pa}$, a duration T=2s, and initial and final frequencies set to $f_{1}=100\;\mathrm{Hz}$ and $f_{2}=10\;\mathrm{kHz}$, respectively. The resulting output signal ${\hat{V}}_{\mathrm{in}}$ is used to drive the DUT, and the corresponding acoustic pressure ${\tilde{p}}_{\mathrm{out}}$ is measured. The SPL of the measured pressure signal is then obtained by deconvolving ${\tilde{p}}_{\mathrm{out}}$ and applying (3). Finally, the SPL associated with ${\tilde{p}}_{\mathrm{out}}$ is compared to the target SPL of $p_{\mathrm{out}}$, as shown in Figure 9.

The two SPL curves closely match across most of the considered frequency range. Up to 5kHz, the equalized SPL curve exhibits a deviation from the target curve, quantified by a Mean Absolute Error (MAE) of approximately 0.5dB. However, near 10kHz, a larger mismatch appears due to the limitations of the linear direct loudspeaker model (and then of its inverse) in accurately reproducing the quality factor of the loudspeaker’s mechanical resonance, resulting in discrepancies of up to 5dB.

**Figure 10:**
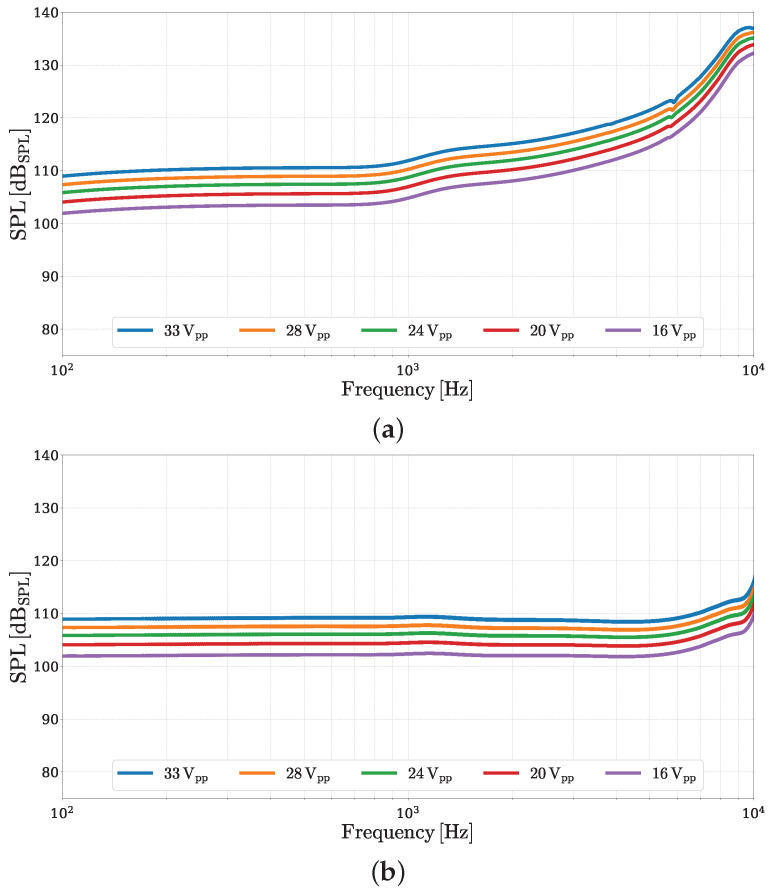
Comparison of frequency-domain SPL curves for different peak-to-peak input voltage levels. (**a**) Measurements with chirps with increasing $V_{\mathrm{pp}}$ values, without equalization. (**b**) Measurements with equalized input signals, each rescaled to have a maximum peak-to-peak amplitude matching the corresponding $V_{\mathrm{pp}}$ value.

In a second experimental validation, we assess the effectiveness of the pre-compensated input signal across different voltage amplitudes. Starting from the previously obtained ${\hat{V}}_{\mathrm{in}}$, we apply a linear rescaling to achieve specific peak-to-peak voltage ($V_{\mathrm{pp}}$) values. This approach serves a dual purpose: first, it ensures that the voltage applied to the MEMS loudspeaker remains within safe operational limits, thus preventing potential damage to the device; second, it enables a fair comparison with the non-equalized SPL measurements by matching the maximum voltage level across the test conditions. The outcomes of this comparison are shown in Figure 10. The selected $V_{\mathrm{pp}}$ values represent a realistic span of operational conditions from moderate to near-maximum driving levels. As illustrated in Figure 10a, the non-equalized SPL curves exhibit a dynamic range exceeding 30dB for each considered $V_{\mathrm{pp}}$ value, whereas the equalized SPL curves, displayed in Figure 10b, remain significantly more controlled, with deviations from flatness not exceeding 5dB across the entire the frequency range. These results underscore the effectiveness of inverse-model-based equalization in consistently flattening the loudspeaker’s frequency response across varying voltage amplitudes.

### 4.2. Discussion

Beyond its effectiveness in flattening the loudspeaker frequency response, the proposed inverse-model-based equalization method also has a notable impact on the system’s nonlinear behavior. Building on the results discussed in the previous subsection, where equalized and non-equalized SPL curves were compared across various peak-to-peak input voltages, we extend the analysis to evaluate how the equalization process affects distortion performance. The proposed equalization strategy works by compensating for the frequency-dependent behavior of the loudspeaker. To achieve a flat SPL, it often requires reducing the input signal amplitude at higher frequencies, where the loudspeaker’s acoustic response tends to be more pronounced due to the presence of its mechanical resonance peak. This amplitude rescaling, although resulting in a lower acoustic output, helps with minimizing the impact of nonlinearities.

**Figure 11:**
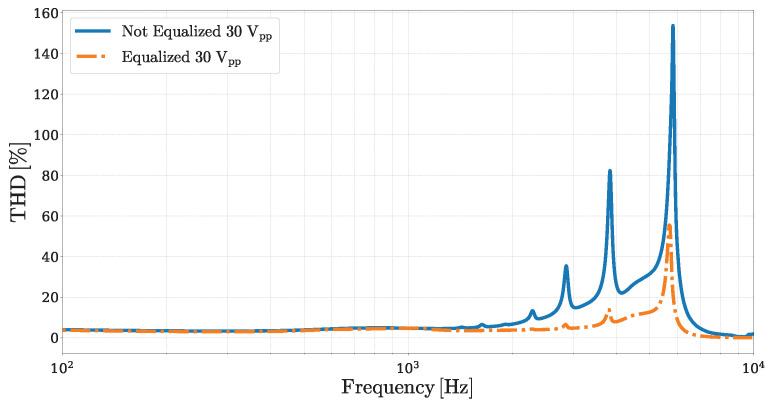
Comparison of frequency-domain THD ratio curves: THD measurements obtained by driving the MEMS loudspeaker with a $30\;V_{\mathrm{pp}}$ signal without any equalization (solid blue curve) are compared to THD measurements obtained using an equalized input signal (orange dash-dotted curve), rescaled to have a maximum peak-to-peak amplitude matching the corresponding $30\;V_{\mathrm{pp}}$.

In piezoelectrically actuated MEMS loudspeakers, distortion can originate from multiple sources, including the intrinsic hysteretic nature of the piezoelectric transducer material and mechanical nonlinearities due to large diaphragm displacements [5,19,31]. By attenuating the driving signal in frequency regions where these effects are more pronounced, the equalization method naturally limits their influence. Figure 11 illustrates a comparison between the Total Harmonic Distortion (THD) ratio curves obtained for the non-equalized and equalized cases, both driven with a maximum peak-to-peak voltage of $30\;V_{\mathrm{pp}}$. In the lower-frequency region, where the input amplitude remains relatively high even after equalization, the THD remains similar between the two conditions. However, at higher frequencies, the equalized system exhibits a significant reduction in the THD, particularly in the regions where the original system shows strong distortion peaks. It is important to note that this improvement in linearity is achieved at the expense of radiating power. This trade-off between linearity and output level is intrinsic to any method that relies on dynamic range shaping to mitigate distortion.

## 5. Conclusions

In this manuscript, we presented a model-based equalization approach to compensate for the non-ideal frequency response of a MEMS loudspeaker designed for in-ear applications. The method leverages a nullor-based inverse model derived from a proposed MEMS loudspeaker equivalent circuit model, digitally implemented using WDFs. After validating the accuracy of the equivalent circuit model against experimental acoustic measurements, we employed its inverse to pre-process the loudspeaker’s input signal to achieve a flat target frequency response. The experimental results demonstrated that the inverse-model-based equalization effectively flattened the loudspeaker’s SPL response, maintaining only small deviations from the target response across the considered frequency range. In addition to equalization, we showed that the proposed pre-processing approach contributed to a reduction in the THD, particularly at higher frequencies, although at the cost of a reduced acoustic output level.

Future work might aim to extend the method to explicitly address the nonlinear behavior of MEMS loudspeakers. In particular, considering a nonlinear MEMS loudspeaker model into the inverse system design within the DIDC framework would enable the pre-processing chain to compensate not only the frequency response’s non-idealities but also nonlinear distortions. By accurately modeling and inverting the device’s nonlinear characteristics, the processing strategy could be adapted to effectively linearize the loudspeaker’s behavior.

## Figures and Tables

**Table 1:** Values of the electro-mechano-acoustic circuital parameters in Figure 2.

Parameter	Value	Unit
$R_{e}$	1	Ω
$C_{p}$	$3.50\times 10^{-8}$	F
α	6.61×10−42	$N\cdot V^{-1}$
$R_{m}$	$8.60\times 10^{-4}$	$N\cdot s\cdot m^{-1}$
$M_{m}$	$1.31\times 10^{-7}$	kg
$C_{m}$	$2.17\times 10^{-3}$	$m\cdot N^{-1}$
$S_{\mathrm{eff}}$	$4.08\times 10^{-6}$	$m^{2}$
$C_{\mathrm{bc}}$	$7.05\times 10^{-12}$	$\mathrm{Pa}\cdot m^{-3}$
$R_{\mathrm{slit}}$	$6.55\times 10^{8}$	$\mathrm{Pa}\cdot s\cdot m^{-3}$

**Table 2:** Values of the IEC 60318-4 ear simulator equivalent circuit model parameters with additional damping terms.

Parameter	Value	Unit
$R_{1}$	$1.66\times 10^{7}$	$\mathrm{Pa}\cdot s\cdot m^{-3}$
$R_{2}$	$5.57\times 10^{7}$	$\mathrm{Pa}\cdot s\cdot m^{-3}$
$R_{3}$	$1.66\times 10^{7}$	$\mathrm{Pa}\cdot s\cdot m^{-3}$
$R_{4}$	$2.80\times 10^{7}$	$\mathrm{Pa}\cdot s\cdot m^{-3}$
$R_{5}$	$1.66\times 10^{7}$	$\mathrm{Pa}\cdot s\cdot m^{-3}$
$L_{1}$	82.9	$\mathrm{Pa}\cdot s^{2}\cdot m^{-3}$
$L_{2}$	9400	$\mathrm{Pa}\cdot s^{2}\cdot m^{-3}$
$L_{3}$	130.3	$\mathrm{Pa}\cdot s^{2}\cdot m^{-3}$
$L_{4}$	983.8	$\mathrm{Pa}\cdot s^{2}\cdot m^{-3}$
$L_{5}$	133.4	$\mathrm{Pa}\cdot s^{2}\cdot m^{-3}$
$C_{1}$	$8.00\times 10^{-13}$	$\mathrm{Pa}\cdot m^{-3}$
$C_{2}$	$2.34\times 10^{-12}$	$\mathrm{Pa}\cdot m^{-3}$
$C_{3}$	$1.50\times 10^{-12}$	$\mathrm{Pa}\cdot m^{-3}$
$C_{4}$	$2.73\times 10^{-12}$	$\mathrm{Pa}\cdot m^{-3}$
$C_{5}$	$1.52\times 10^{-12}$	$\mathrm{Pa}\cdot m^{-3}$

## Data Availability

The raw data supporting the conclusions of this article will be made available by the authors upon request.

## Abbreviations

The following abbreviations are used in this manuscript:

DFT	Discrete Fourier Transform
DIDC	Direct-Inverse-Direct Chain
DSP	Digital Signal Processing
DUT	Device Under Test
EPI	Epitaxial
FIR	Finite Impulse Response
IIR	Infinite Impulse Response
LEM	Lumped-Element Model
MAE	Mean Absolute Error
MEMS	Micro-Electro-Mechanical Systems
PCB	Printable Circuit Board
PZT	Lead Zirconate Titanate
SPL	Sound Pressure Level
THD	Total Harmonic Distortion
TIPC	Target-Inverse-Physical Chain
WD	Wave Digital
WDF	Wave Digital Filter
